# Time‐Dependent Changes in Effects of Butyrate on Human Gingival Fibroblasts

**DOI:** 10.1002/cre2.70120

**Published:** 2025-04-24

**Authors:** Haruki Otani, Jumpei Washio, Aoi Kunitomi, Satoko Sato, Yuki Abiko, Shiori Sasaki, Kazumasa Ohashi, Satoru Yamada, Nobuhiro Takahashi

**Affiliations:** ^1^ Division of Oral Ecology and Biochemistry Tohoku University Graduate School of Dentistry Sendai Japan; ^2^ Division of Periodontology and Endodontology Tohoku University Graduate School of Dentistry Sendai Japan; ^3^ Laboratory of Molecular and Cellular Biology, Department of Molecular and Chemical Life Sciences Tohoku University Graduate School of Life Sciences Sendai Japan

**Keywords:** fibroblast, microbiology, morphology, periodontal disease

## Abstract

**Objectives:**

Butyrate is one of major metabolites of periodontitis‐associated bacteria and often detected in periodontal pockets. Butyrate has been considered to affect human gingival fibroblasts (HGFs); however, there was no information on its long‐term effect as occurs in periodontitis. Therefore, this study aimed to evaluate the time‐dependent effects of butyrate on HGFs.

**Material and Methods:**

The effects of butyrate on HGF proliferation, apoptosis, cell morphology, glucose metabolic activity, butyrate metabolic activity, and cell migration ability were evaluated by cell counting, DNA electrophoresis, cell staining, pH‐stat system, HPLC, and scratch test, respectively.

**Results:**

HGF proliferation was temporarily inhibited by 5–10 mM butyrate (*p* < 0.05); however, it resumed at 24 h with morphological changes from spindle to slightly widened (*p* < 0.05). HGFs cultured with 10 mM butyrate for 12–24 h shifted the glucose metabolic pathway from oxidative phosphorylation to glycolysis (*p* < 0.05), and increased butyrate consumption, which returned to control levels over 24 h. HGF migration ability tended to decrease at 72 h.

**Conclusions:**

HGF cell proliferation and glucose/butyrate metabolism were temporarily inhibited by butyrate and then recovered in a time‐dependent manner, accompanied by changes in cell morphology. These time‐dependent effects may help to understand the role of butyrate in the pathology of periodontitis.

## 1 Introduction

Periodontitis is a chronic inflammatory disease caused by accumulated oral biofilm, and it is well‐known that periodontitis‐associated bacteria, such as *Porphyromonas gingivalis* and *Fusobacterium nucleatum*, are closely associated with this disease. These bacteria exhibit LPS and fimbria on membrane components and extracellularly produce various proteases and metabolites such as short‐chain fatty acids (SCFAs), sulfide, and ammonia. These substances not only directly damage periodontal tissues (Cueno and Ochiai 2016), but also indirectly contribute to bone resorption through their effects on immune cells (Mysak et al. 2014).

Butyrate, one of the SCFAs, is a major metabolite produced when periodontitis‐associated bacteria metabolize proteins and amino acids derived from gingival crevicular fluid, blood, and periodontal tissue (Takahashl et al. 1997; Takahashi et al. 2000; Takahashi 2015). Butyrate is frequently detected at 3–4 mM in the gingival sulcus of healthy individuals, and approximately 10 mM in the periodontal pockets of patients with periodontitis, depending on severity (Tonetti et al. 1987; Niederman et al. 1997). Previous studies reported that butyrate inhibits gingival cells (Sorkin and Niederman 1998; Tsuda et al. 2010; Kurita‐Ochiai et al. 2008; Pöllänen et al. 1997; Chang et al. 2013). Butyrate was reported to inhibit cell proliferation, induce apoptosis with the production of inflammatory cytokines (Shirasugi et al. 2018), and promote the production of matrix metalloproteinases of human gingival fibroblasts (HGFs) (Nakagawa et al. 2021). In addition, butyrate was reported to induce death of immune cells (Kurita‐Ochiai et al. 1997, 1998, 2000, 2001). These studies suggest that butyrate contributes to the onset and/or progression of periodontitis.

In a recent study, however, a lower concentration (about 1 mM) of butyrate was reported to suppress dendritic cell inflammation in periodontal tissue and alleviates periodontitis (Wu et al. 2023). Furthermore, butyrate is also known to be beneficial for intestinal epithelial cells; butyrate produced by the gut microbiota is utilized as an energy source (Hamer et al. 2008; Donohoe et al. 2011) and increases the cell integrity (Kelly et al. 2015). In addition, considering that butyrate is present in gingival sulci and periodontal pockets for a long period of time, a long‐term effect of butyrate should be evaluated. Hence, it is hypothesized that even in the oral cavity, the effect of butyrate may vary depending on the concentration and duration of action.

Therefore, the present study aimed to comprehensively evaluate the effects of butyrate on HGF functions such as proliferation, cell death, morphology, glucose metabolism, butyrate consumption, and migration ability, and further clarify the concentration‐dependent and time‐dependent effect of butyrate.

## 2 Materials and Methods

### 2.1 Cell Lines and Culture Conditions

A human gingival fibroblast cell line (HGF) (PCS‐201‐018, ATCC, Virginia, USA) was used. HGF were cultured in Dulbecco's Modified Eagle's medium DMEM (10‐103‐CVR, Corning, NY, USA) supplemented with 2 mM l‐glutamine solution (Wako Pure Chemical Industries, Osaka, Japan), 10% heat‐inactivated FBS fetal bovine serum (Thermo Fisher Scientific, Waltham, Massachusetts, USA), 100 mg/mL streptomycin solution, and 100 U/mL penicillin solution at 37°C under 5% CO_2_. The cells were collected at 80%–90% confluence, then suspended in physiological saline (cell suspension), and stored at 4°C until use.

### 2.2 Cell Proliferation Assay

HGF were seeded at 2.5 × 10⁵ cells/dish and cultured overnight. Then, HGF were cultured with sodium butyrate (final concentrations: 0.5, 5, and 10 mM) for 24, 72, and 120 h, and the cell number was counted by Countless TM II FL (Thermo Fisher Scientific, Waltham, MA, USA).

### 2.3 Detection of DNA Fragmentation

To investigate the possibility of apoptosis, intracellular DNA was extracted from HGF cultured with sodium butyrate (final concentration: 10 mM) for 12, 24, or 72 h, and the presence of DNA fragmentation was checked by electrophoresis using 2% agarose gel and ApopLadder Ex (Takara Bio Inc., Shiga, Japan).

### 2.4 Cell Morphology Assay

Cell morphology was analyzed by immunofluorescence staining. HGF were seeded at 5.0 × 10⁵ cells in each well of a 6‐well plate and incubated overnight, and then cultured with 10 mM butyrate for 12, 24, or 72 h. Cell fixation was then performed with 4% paraformaldehyde for 20 min, and permeabilization was carried out using 0.5% Triton. After washing with PBS, the cells were blocked with 5% FBS in PBS for 30 min. After washing with PBS again, the fixed cells were incubated with DAPI and Phalloidin for 10 min at room temperature and washed with PBS. Cells were observed using a DMI 8 fluorescence microscope (Leica Microsystems, Wetzlar, Germany) equipped with a PL Apo 20× oil objective lens (0.8 NA). In addition, ImageJ software was used to calculate the area and aspect ratio (length/width ratio) of cells.

### 2.5 Measurement of Glucose Metabolic Activity Using pH‐Stat System

The glucose metabolic activity of HGF was measured using an automatic acid titrator: pH‐stat system (AUTO pH STAT: Model AUT‐501, TOA DKK, Aichi, Japan). This system can estimate the metabolic activity of cells based on acid production from glucose. Sodium butyrate (final concentration: 5 or 10 mM) was added into the dish containing HGF culture for the indicated time, and each of the cell suspensions (3.0 × 10⁶ cells/500 µL) was prepared as previously described. The reaction mixture containing 500 µL of the cell suspension and 4.25 mL of physiological saline was preincubated at 37°C for 5 min. Then, 0.25 mL of 100 mM glucose was added to the mixture (final concentration: 10 mM), and the acid‐producing activity at pH 7.45 was monitored for 10 min using the pH‐stat system (Morishima et al. 2017; Shinohara et al. 2021).

### 2.6 Lactate Assay

On measurement with the pH‐stat system, a small amount of the reaction mixture was collected immediately after adding glucose and directly after the end of measurement and analyzed with HPLC (Shimadzu Prominence LC‐20AD; Shimadzu Corporation, Kyoto, Japan) to determine the levels of organic acids produced through glucose metabolism in the sample. Before analysis with HPLC, the samples were filtered through a polypropylene membrane (pore size: 0.20 µm; Tokyo Roshi Ltd., Tokyo, Japan) (Manome et al. 2019; Han et al. 2021). No organic acids other than lactate were detected.

### 2.7 Estimation of Glucose Consumption and ATP Production

Amounts of ATP production and glucose consumption with the addition of 10 mM butyrate were estimated from levels of lactate and CO_2_ production. The amount of CO_2_ produced was calculated from the amount of acid produced other than lactate measured using the pH stat system, as described above. CO_2_ reacts with H_2_O to form H_2_CO_3_. Since pKa of H_2_CO_3_ is 6.3, Henderson–Hasselbalch's equation calculates that at pH 7.45, 93.4% of H_2_CO_3_ dissociates into H^+^ + HCO_3_^‐^. Therefore, the actual amount of CO_2_ produced is calculated to be 1.07 times the estimated amount of CO_2_ produced. In general, when one molecule of glucose is fermented through glycolysis and there is conversion of pyruvate to lactate, two molecules of lactate and two molecules of ATP are produced, whereas, when one molecule of glucose is fermented through OXPHOS (glycolysis, TCA cycle, and electron transfer system), six molecules of CO_2_ and about 36 molecules of ATP are produced. Therefore, amounts of glucose consumption were estimated from [lactate production × 1/2 + carbon dioxide production × 1/6], and the amounts of ATP production were similarly estimated from [lactate production × 1 + carbon dioxide production × 6] according to these proposed metabolic pathways.

### 2.8 Measurement of Butyrate Consumption

HGF were harvested after 12, 24, 48, and 72 h of culture with 10 mM sodium butyrate, and each of the cell suspensions (3.0 × 10⁶ cells/500 µL) was prepared. A total of 250 µL of sodium butyrate solution (final concentrations: 5 and 10 mM) was added to cell suspensions. To measure butyrate consumption by HGF, a small amount of the reaction mixture was taken immediately after the addition of butyrate and at 10 min after its addition and analyzed by HPLC as described above. The butyrate consumption for 10 min was calculated from the changes in butyrate concentration.

### 2.9 Migration Assay

The effect of butyrate on migration was analyzed as described previously (Buranasin et al. 2018). After culturing HGF to 80% confluence, 10 mM butyrate was added to the culture medium for 12, 24, or 72 h. Then, a 0.6 ± 0.05‐mm‐wide layer of cells was scratched and removed from the culture dish using a OneTouch Tips 200 µL barrier tip (Sorenson, Salt Lake City, USA). After washing the culture dish with PBS and filling it with fresh cell culture medium without butyrate, cell migration was observed every 24 h using a microscope (CK30, OLYMPAS, Tokyo, Japan). ImageJ software was used to calculate cell migration rates.

### 2.10 Statistical Analysis

A paired t‐test was used for comparisons between two groups, and Tukey's test was used for comparisons among three or more groups. A *p*‐value of less than 0.05 was defined as significant. The software applied for these analyses was Excel or Stat Flex Ver. 7 (Artec Co. Ltd., Osaka, Japan).

## 3 Results

### 3.1 The Effect of Butyrate on Cell Proliferation

**Figure 1:**
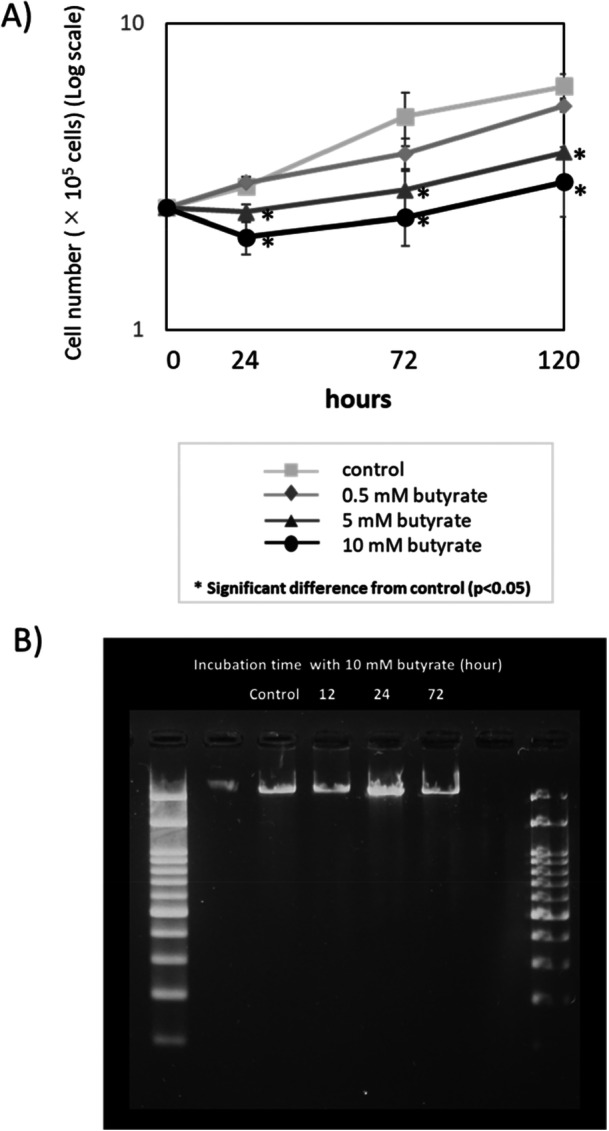
Effects of butyrate on cell proliferation and apoptosis. (A) HGF were initially seeded at 2.5 × 10⁵ cells/dish and cultured with sodium butyrate (final concentration: 0.5, 5, and 10 mM). Cell numbers were counted after 24, 72, and 120 h. *Significant difference from control (*p* < 0.05). (B) Electrophoretic images for the detection of DNA fragments. From left to right: ladder marker (100 bp), blank, Control, 12, 24, 72 h, blank, same ladder marker. DNA fragmentation was not detected in any of the groups.

Coexistence with 5 or 10 mM butyrate for 24 h significantly decreased the number of HGF; however, the cells began to proliferate after 24 h, with the proliferation rate reaching a similar level to that in the control (Figure 1A). DNA fragmentation indicative of the possibility of cell apoptosis was not detected under any of the conditions (Figure 1B).

### 3.2 The Effects of Butyrate on Morphology

**Figure 2:**
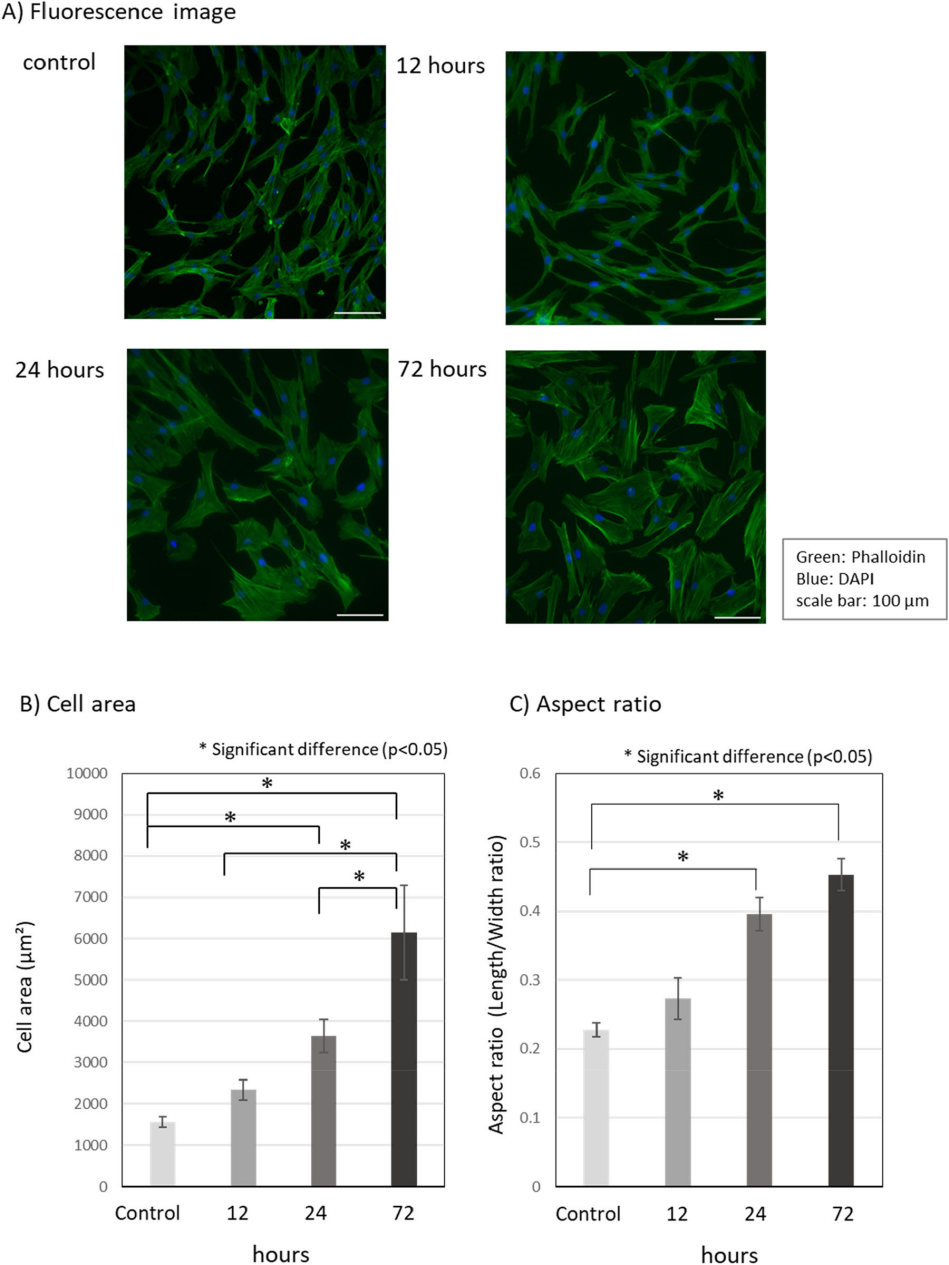
Effects of butyrate on cell morphology. Morphology of HGF cultured with butyrate for indicated times (0–72 h). (A) Fluorescence image of HGF cultured with butyrate. Green: Phalloidin. Blue: DAPI. Scale bar: 100 µm. (B) Cell area (µm²), average ± standard deviation calculated from the value of 50 cells. (C) Aspect ratio (length/width ratio) of HGF cultured with butyrate was measured using ImageJ software, average ± standard deviation calculated from the value of 50 cells. *Significant difference from control (*p* < 0.05).

The morphology of HGF was changed by butyrate over time (Figure 2A). The cell area significantly increased in a time‐dependent manner (Figure 2B). In addition, the cell morphology changed in a time‐dependent manner from a spindle shape, which is the typical fibroblast morphology, to a slightly widened cell shape. The aspect ratio, which represents the ratio of length to width, significantly increased in a time‐dependent manner (Figure 2C). The results at 120 h were not different from those at 72 h (data not shown), suggesting that morphological changes in HGF reach a maximum at 72 h.

### 3.3 The Effects of Butyrate on the Glucose Metabolic Activity and Metabolic Pattern of HGF

**Figure 3:**
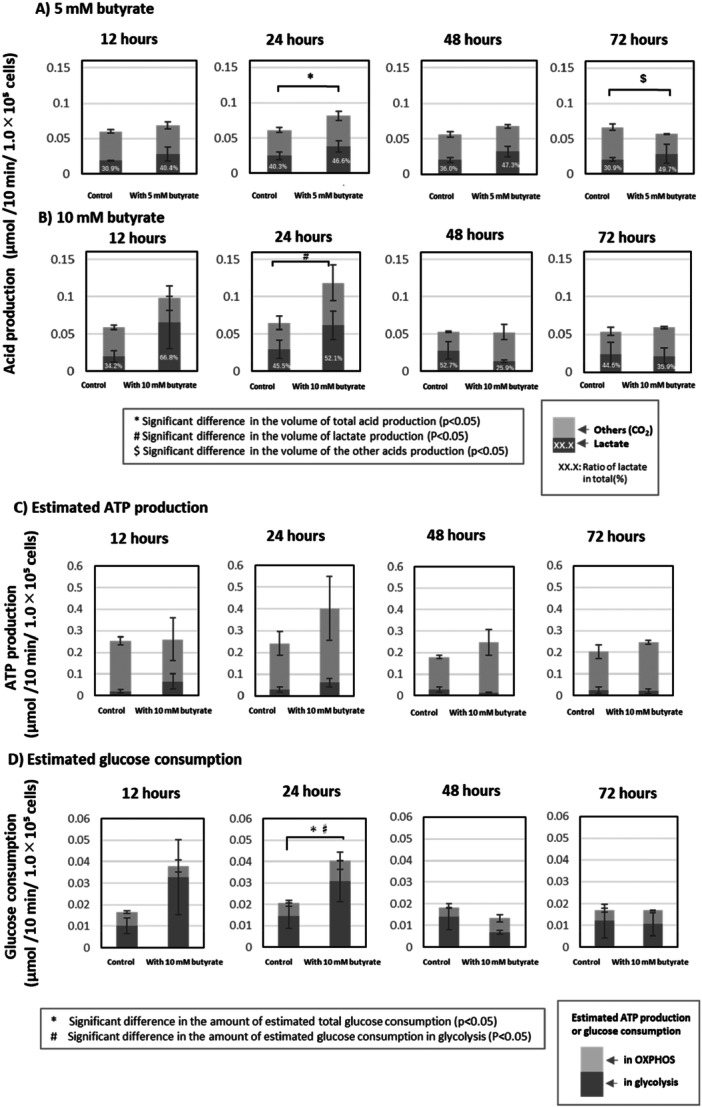
Effects of butyrate on glucose metabolism. (A, B) The amount of acid production (µmol/10 min/1.0 × 10^5^ cells) due to glucose metabolism of HGF cultured with (A) 5 mM or (B) 10 mM butyrate for the indicated times (12–72 h). The height of the bar indicates the acid production under each condition. Light and dark gray bars indicate the amount of lactate and other acids, respectively. Error bars indicate the standard deviation for each amount of lactate and other acids. *Significant difference in the ratio of lactate in total acid production (*p* < 0.05). #Significant difference in volume of lactate production (*p* < 0.05). $Significant difference in volume of other acids produced (*p* < 0.05). (C, D) The estimated amount of ATP production (C) and glucose consumption (D) (µmol/10 min/1.0 × 10^5^ cells) in the cells cultured with 10 mM butyrate for the indicated times (12–72 h). The height of the bar indicates each amount under each condition. Light and dark gray bars indicate the amount of ATP production or glucose consumption in OXPHOS and glycolysis, respectively. Error bars indicate the standard deviation for each production of lactate and other acids. Error bars indicate the standard deviation for estimated ATP production or glucose consumption in each of glycolysis or OXPHOS. *Significant difference in total amount of glucose consumption (*p* < 0.05). #Significant difference in the estimated amount of glucose consumption in glycolysis (*p* < 0.05).

In HGF cultured with 5 mM butyrate for 12–48 h, the total acid production tended to increase, whereas in cells cultured for 72 h it decreased (Figure 3A). The total acid production of HGF cultured with 10 mM butyrate for 12 or 24 h clearly increased; however, after 48 and 72 h the total acid production had returned to the control level (Figure 3B).

To estimate the pattern of glucose metabolism, i.e., the dependence of the glycolysis‐lactate production pathway and glycolysis‐TCA cycle‐electron transfer system pathway (oxidative phosphorylation system, OXPHOS), the ratio of lactate to total acid production was calculated. In the present study, since only lactate was detected with HPLC (data not shown), the acid produced other than lactate was considered to be bicarbonate derived mainly from CO_2_ produced through OXPHOS. The amount and ratio of lactate tended to increase in cells cultured with 5 mM butyrate for 12–48 h. Similarly, the amount and ratio of lactate tended to increase in cells cultured with 10 mM butyrate for 12 h, and the amount of lactate increased significantly for 24 h. On the other hand, the amount of lactate decreased when cells were cultured with 10 mM butyrate for 48 h. Finally, both the amount and ratio of lactate returned to the same levels as in the control after 72 h (Figure 3A, B).

### 3.4 The Effects of Butyrate on ATP Production and Glucose Consumption

In HGF cultured with 10 mM butyrate for 24 h, ATP production tended to increase, especially the production derived from OXPHOS; however, in cells after 48 and 72 h, it had returned to the control level (Figure 3C). Furthermore, the percentage of glycolysis‐derived ATP production was lower in cells at 24, 48, and 72 h compared with 12 h.

Similarly, in cells cultured with 10 mM butyrate for 12 h, glucose consumption tended to increase, and increased significantly in cells after 24 h, especially consumption in glycolysis; however, in cells after 48 or 72 h, it had returned to the control level (Figure 3D). Furthermore, the rate of glucose consumption in glycolysis was lower in cells at 48 and 72 h compared with 12 and 24 h.

### 3.5 Butyrate Consumption by HGF

**Figure 4:**
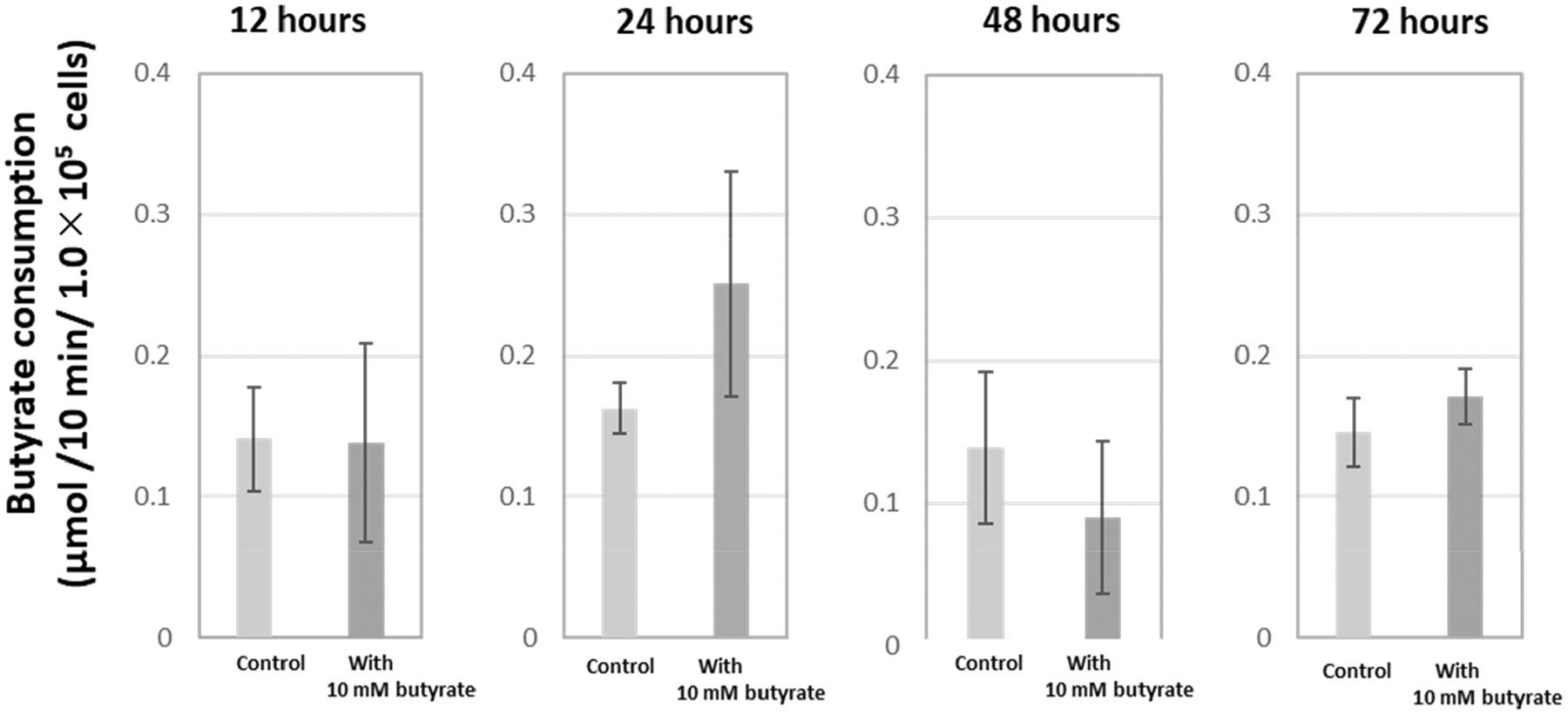
The amount of butyrate consumed by HGF. The amount of butyrate consumption (µmol/10 min/1.0 × 10^5^ cells) by HGF cultured with 10 mM butyrate for the indicated times (12–72 h).

The activity of butyrate consumption was at the same level as in the control in cells cultured with butyrate for 12 h, but increased when cultured for 24 h. At 72 h, the consumption activity had returned to the control level (Figure 4).

### 3.6 The Effect of Butyrate on Cell Migration

**Figure 5:**
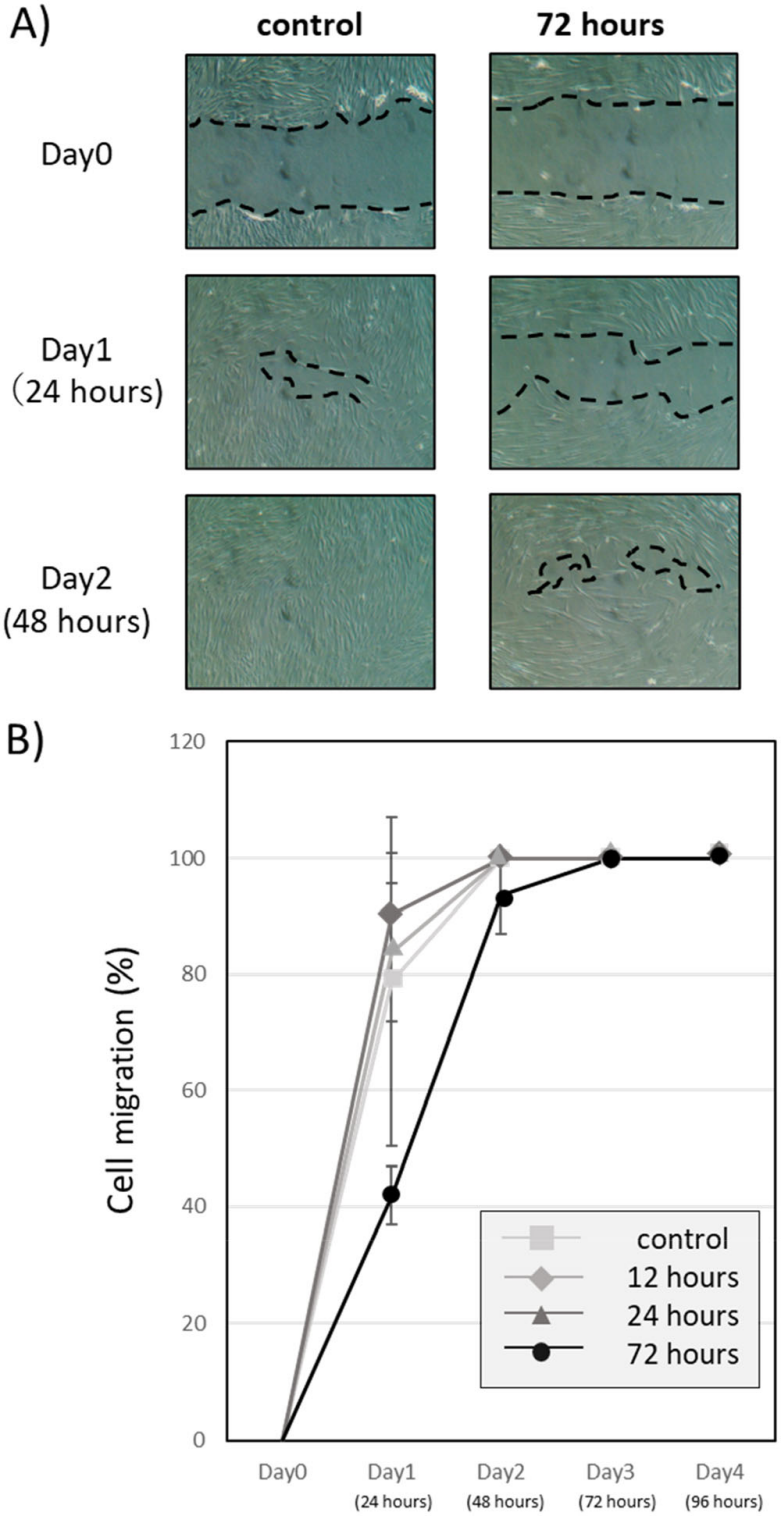
Effects of butyrate on cell migration. (A) Time‐dependent microscopic images (0–48 h) of cell migration in HGF cultured with 10 mM butyrate for 0 or 72 h. A 0.6 ± 0.05‐mm‐wide layer of cells was scratched off and removed from the culture dish. Areas surrounded by dotted lines: areas where cells were scratched (Day 0), areas where cell migration was not observed (Days 1 and 2). (B) The percentage area recovered by cell migration at 0, 12, 24, and 72 h, in HGF cultured with 10 mM butyrate for the indicated times (0‐72 h).

Migration of HGF cultured with sodium butyrate was inhibited in a culture time‐dependent manner, especially at 72 h, although not significantly. In all groups, wounds had completely closed by Day 4 (Figure 5A, B).

## 4 Discussion

In this study, we cultured HGF with 5–10 mM butyrate, which is clinically observed in periodontal pockets (Tonetti et al. 1987; Niederman et al. 1997), and evaluated the time‐dependent effects. The presence of butyrate in the culture medium initially resulted in a decrease in cell number, i.e., inhibition of proliferation. However, as the incubation time increased, the cell proliferation gradually recovered (Figure 1A). Furthermore, DNA fragmentation indicating possible apoptosis was not detected, suggesting that butyrate inhibited HGF proliferation by inducing cell death other than apoptosis (Figure 1B). In previous studies, when primary HGF were cultured with up to 15 mM butyrate for 72 h, apoptosis induction was shown by positive Annexin V staining and the appearance of caspase 8, 9, and 3/7 activity (Shirasugi et al. 2017), suggesting that a higher concentration of butyrate may lead to apoptosis. On the other hand, butyrate (1.25–5 mM for 48 h) did not induce cell death in HGF derived from healthy periodontal tissue (Kurita‐Ochiai et al. 2008), further supporting that a lower concentration of butyrate does not induce apoptosis of healthy HGF as observed in our study. Although in the present study, we inferred that apoptosis did not occur based on DNA fragmentation data alone, it may be important to accurately confirm the presence of apoptosis using more sensitive methods such as Annexin V staining and caspase activity. If the presence of apoptosis is not observed even using these sensitive methods, other cell deaths events such as pyroptosis, autophagy, ferroptosis, and cuproptosis (Bertheloot et al. 2021; Zheng et al. 2025) may be considered.

Furthermore, the recovery of HGF proliferation was newly demonstrated by our study (Figure 1A), indicating the time‐dependent response of HGF to butyrate. These findings suggest that the response of HGF to butyrate is biphasic in time: a transient impairment of cell proliferation immediately after coexistence with butyrate (5–10 mM for 24 h), followed by the recovery of cell proliferation, that is, the acquisition of tolerance to butyrate. Thus, we furthered our comprehensive evaluation of cell morphology, metabolic activity, and migration ability.

In the present study, the culture with butyrate resulted in time‐dependent changes in HGF morphology from the HGF‐specific spindle shape to a slightly widened shape (Figures 2A, 2C), and an up to approximately fourfold increase in cell area (Figure 2B). Jeng et al. also reported that culture with 8–24 mM butyrate for 5 days caused changes in cell morphology (Jeng et al. 1999), although they did not measure the cell area or calculate the aspect ratio, being consistent with our results. Amoedo et al. reported that sodium butyrate induces morphological changes of lung cancer H460 cells and suggested that alterations in cell shape may be related to cytoskeleton reorganization since the treatment of cells with sodium butyrate led to a marked redistribution of F‐actin (Amoêdo et al. 2011). Mathew et al. also reported that inactivation of Akt by butyrate differentially affects FOXO transcription factors, caspase 3 and PARP, mTOR, and GSK3, inducing proliferation arrest and altering cellular size and morphology (Mathew et al. 2019). However, further research is required to elucidate the entire mechanism of the effect of butyrate on HGF.

Next, the effect of butyrate on glucose metabolism was evaluated. For glucose metabolism of HGF, culture with 10 mM butyrate for 12–24 h resulted in a shift in the glucose metabolic pathway from OXPHOS to lactate production at 12–24 h with an increase in acid production (i.e., glucose metabolic activity), but then returned to that noted in controls (Figure 3A, B). The glucose metabolic activity and pathway closely influence cellular ATP production, being due to the efficiency of ATP production as described in Materials and Methods. Considering the above, although glucose metabolic activity was increased at 12 h and the metabolic pathway shifted to lactate production (Figure 3B), the estimated ATP production was at the same level as in the control. However, the estimated ATP production at 24 h was about 1.6 times higher than in the control, because the glucose metabolic pathway shifted to OXPHOS again (Figure 3C). At the same time, glucose consumption also increased significantly at 24 h (Figure 3D). Such changes in ATP supply may directly affect cell proliferation, which may have led to the initial decrease in cell number and subsequent recovery of proliferation during culture with butyrate (Figure 1), although further studies are needed to determine how changes in ATP supply affect various cellular functions other than proliferation. Previous studies suggest that an increase in cellular ATP production promotes actin remodeling of the cytoskeleton, which is involved in the maintenance and alteration of cell morphology (DeWane et al. 2021; Liu et al. 2022). Actin remodeling consists of the reorganization of F‐actin by polymerization and depolymerization of G‐actin, and polymerization occurs in an ATP‐dependent manner (Korn et al. 1987). Therefore, the increase of ATP production through a transient increase in glucose metabolism due to coexistence with butyrate may contribute to cell morphological changes and area increases (Figure 2).

In addition, the present study demonstrated that similar to intestinal cells (Hamer et al. 2008), HGF constantly consumed butyrate, but consumption was increased on 24‐h culture with butyrate (Figure 4), which may contribute to the increased cellular ATP production on 24‐h culture with butyrate, followed by the recovery of cell proliferation and changes in cell morphology.

In the present study, butyrate tended to inhibit HGF migration, but not significantly (Figures 5A, 5B). Butyrate was shown to suppress colorectal cancer cell motility by inhibiting HDAC activity and decreasing phosphorylation of Akt1 and ERK1/2 (Li et al. 2017), suggesting a similar mechanism is involved in HGF. However, the inhibitory effect of butyrate on HGF was weaker than that on cancer cells, and the mechanism needs to be further studied.

Furthermore, although the scratches eventually closed by Day 4, but butyrate clearly delayed that closure. This delay may be clinically significant because during the delayed closure, the wound site may induce new infection and the spread of inflammation, leading to more severe periodontitis. Furthermore, in this experiment, scratch healing was observed under butyrate‐free conditions, which is different from the actual gingival tissue environment where butyrate is always present. When wound healing occurs in an environment where butyrate is always present, as in real gingival tissue, wound healing would be expected to be more prolonged, and the clinical impact may be greater. Therefore, considering that butyrate is always present in periodontal tissue, routine inflammation control, such as tooth brushing, may be clinically important. Although future research is needed to further determine the impact of these changes on wound healing, it is thought that the removal and control of butyrate or butyrate‐producing bacteria will be important in the treatment of periodontitis in the future.

## 5 Conclusion

Coexistence with butyrate temporarily altered cell proliferation and glucose metabolism of HGF, but these functions were restored in a time‐dependent manner to levels equivalent to those of untreated cells. On the other hand, HGF cultured with butyrate also led to novel alterations, a significant change in cell morphology for over 24 h‐culture and a slight inhibition on cell migration ability at 72 h. These diverse effects of butyrate may contribute to understanding the role of butyrate in the pathology of periodontitis. Until now, butyrate was recognized as contributing to the onset and/or progression of periodontitis; however, recently it has been reported that butyrate suppresses dendritic cell inflammation in periodontal tissue and alleviates periodontitis (Wu et al. 2023). In addition, butyrate has long been known to be beneficial to intestinal epithelial cells (Hamer et al. 2008; Donohoe et al. 2011; Kelly et al. 2015). This study suggests that there is a phenomenon that has been overlooked in short‐term studies – that cells adapt to butyrate over time – which may fill in a gap in our current knowledge about the effects of butyrate. Given these perspectives, it is important to further study the effects of butyrate on host cells and tissues.

## Author Contributions

All authors contributed to the present study. Haruki Otani contributed to design, acquisition, and analysis, and drafted the manuscript. Jumpei Washio contributed to design, acquisition, and analysis, drafted the manuscript, and critically reviewed it. Aoi Kunitomi, Kazumasa Ohashi, Satoko Sato, Yuki Abiko, and Shiori Sasaki contributed to acquisition and analysis. Satoru Yamada and Nobuhiro Takahashi contributed to conception, design, analysis, and interpretation, and critically reviewed the manuscript.

## Conflicts of Interest

The authors declare no conflicts of interest.

## Data Availability

The data that support the findings of this study are available from the corresponding author upon reasonable request.

## References

[cre270120-bib-0001] Amoêdo, N. D. , M. F. Rodrigues , P. Pezzuto , et al. 2011. “Energy Metabolism in H460 Lung Cancer Cells: Effects of Histone Deacetylase Inhibitors.” PLoS One 6, no. 7: e22264. 21789245 10.1371/journal.pone.0022264 PMC3138778

[cre270120-bib-0002] Bertheloot, D. , E. Latz , and B. S. Franklin . 2021. “Necroptosis, Pyroptosis and Apoptosis: An Intricate Game of Cell Death.” Cellular & Molecular Immunology 18, no. 5: 1106–1121. 33785842 10.1038/s41423-020-00630-3 PMC8008022

[cre270120-bib-0003] Buranasin, P. , K. Mizutani , K. Iwasaki , et al. 2018. “High Glucose‐Induced Oxidative Stress Impairs Proliferation and Migration of Human Gingival Fibroblasts.” PLoS One 13, no. 8: e0201855. 30092096 10.1371/journal.pone.0201855 PMC6084939

[cre270120-bib-0004] Chang, M. C. , Y. L. Tsai , Y. W. Chen , et al. 2013. “Butyrate Induces Reactive Oxygen Species Production and Affects Cell Cycle Progression in Human Gingival Fibroblasts.” Journal of Periodontal Research 48, no. 1: 66–73. 22834967 10.1111/j.1600-0765.2012.01504.x

[cre270120-bib-0005] Cueno, M. E. , and K. Ochiai . 2016. “Re‐Discovering Periodontal Butyric Acid: New Insights on an Old Metabolite.” Microbial Pathogenesis 94: 48–53. 26466516 10.1016/j.micpath.2015.10.006

[cre270120-bib-0006] DeWane, G. , A. M. Salvi , and K. A. DeMali . 2021. “Fueling the Cytoskeleton ‐ Links Between Cell Metabolism and Actin Remodeling.” Journal of Cell Science 134, no. 3: jcs248385. 33558441 10.1242/jcs.248385 PMC7888749

[cre270120-bib-0007] Donohoe, D. R. , N. Garge , X. Zhang , et al. 2011. “The Microbiome and Butyrate Regulate Energy Metabolism and Autophagy in the Mammalian Colon.” Cell Metabolism 13, no. 5: 517–526. 21531334 10.1016/j.cmet.2011.02.018 PMC3099420

[cre270120-bib-0008] Hamer, H. M. , D. Jonkers , K. Venema , S. Vanhoutvin , F. J. Troost , and R. J. Brummer . 2008. “Review Article: The Role of Butyrate on Colonic Function.” Alimentary Pharmacology & Therapeutics 27, no. 2: 104–119. 17973645 10.1111/j.1365-2036.2007.03562.x

[cre270120-bib-0009] Han, S. , Y. Abiko , J. Washio , Y. Luo , L. Zhang , and N. Takahashi . 2021. “Green Tea‐Derived Epigallocatechin Gallate Inhibits Acid Production and Promotes the Aggregation of *Streptococcus mutans* and Non‐Mutans Streptococci.” Caries Research 55, no. 3: 205–214. 34010838 10.1159/000515814

[cre270120-bib-0010] Jeng, J. H. , C. P. Chan , Y. S. Ho , W. H. Lan , C. C. Hsieh , and M. C. Chang . 1999. “Effects of Butyrate and Propionate on the Adhesion, Growth, Cell Cycle Kinetics, and Protein Synthesis of Cultured Human Gingival Fibroblasts.” Journal of Periodontology 70, no. 12: 1435–1442. 10632518 10.1902/jop.1999.70.12.1435

[cre270120-bib-0011] Kelly, C. J. , L. Zheng , E. L. Campbell , et al. 2015. “Crosstalk Between Microbiota‐Derived Short‐Chain Fatty Acids and Intestinal Epithelial HIF Augments Tissue Barrier Function.” Cell Host & Microbe 17, no. 5: 662–671. 25865369 10.1016/j.chom.2015.03.005 PMC4433427

[cre270120-bib-0012] Korn, E. D. , M. F. Carlier , and D. Pantaloni . 1987. “Actin Polymerization and ATP Hydrolysis.” Science 238, no. 4827: 638–644. 3672117 10.1126/science.3672117

[cre270120-bib-0013] Kurita‐Ochiai, T. , K. Fukushima , and K. Ochiai . 1997. “Butyric Acid‐Induced Apoptosis of Murine Thymocytes, Splenic T Cells, and Human Jurkat T Cells.” Infection and Immunity 65, no. 1: 35–41. 8975889 10.1128/iai.65.1.35-41.1997 PMC174553

[cre270120-bib-0014] Kurita‐Ochiai, T. , K. Ochiai , and K. Fukushima . 1998. “Volatile Fatty Acid, Metabolic By‐Product of Periodontopathic Bacteria, Induces Apoptosis in WEHI 231 and RAJI B Lymphoma Cells and Splenic B Cells.” Infection and Immunity 66, no. 6: 2587–2594. 9596720 10.1128/iai.66.6.2587-2594.1998 PMC108242

[cre270120-bib-0015] Kurita‐Ochiai, T. , K. Ochiai , and K. Fukushima . 2000. “Butyric‐Acid‐Induced Apoptosis in Murine Thymocytes and Splenic T‐ and B‐Cells Occurs in the Absence of p53.” Journal of Dental Research 79, no. 12: 1948–1954. 11201044 10.1177/00220345000790120501

[cre270120-bib-0016] Kurita‐Ochiai, T. , K. Ochiai , and K. Fukushima . 2001. “Butyric Acid‐Induced T‐Cell Apoptosis Is Mediated by Caspase‐8 and ‐9 Activation in a Fas‐Independent Manner.” Clinical Diagnostic Laboratory Immunology 8, no. 2: 325–332. 11238216 10.1128/CDLI.8.2.325-332.2001 PMC96057

[cre270120-bib-0017] Kurita‐Ochiai, T. , S. Seto , N. Suzuki , et al. 2008. “Butyric Acid Induces Apoptosis in Inflamed Fibroblasts.” Journal of Dental Research 87, no. 1: 51–55. 18096893 10.1177/154405910808700108

[cre270120-bib-0018] Li, Q. , C. Ding , T. Meng , et al. 2017. “Butyrate Suppresses Motility of Colorectal Cancer Cells via Deactivating Akt/ERK Signaling in Histone Deacetylase Dependent Manner.” Journal of Pharmacological Sciences 135, no. 4: 148–155. 29233468 10.1016/j.jphs.2017.11.004

[cre270120-bib-0019] Liu, G. , J. Li , and C. Wu . 2022. “Reciprocal Regulation of Actin Filaments and Cellular Metabolism.” European Journal of Cell Biology 101, no. 4: 151281. 36343493 10.1016/j.ejcb.2022.151281

[cre270120-bib-0020] Manome, A. , Y. Abiko , J. Kawashima , J. Washio , S. Fukumoto , and N. Takahashi . 2019. “Acidogenic Potential of Oral Bifidobacterium and Its High Fluoride Tolerance.” Frontiers in Microbiology 10: 1099. 31156604 10.3389/fmicb.2019.01099 PMC6532017

[cre270120-bib-0021] Mathew, O. P. , K. Ranganna , J. Mathew , et al. 2019. “Cellular Effects of Butyrate on Vascular Smooth Muscle Cells Are Mediated Through Disparate Actions on Dual Targets, Histone Deacetylase (HDAC) Activity and PI3K/Akt Signaling Network.” International Journal of Molecular Sciences 20, no. 12: 2902. 31197106 10.3390/ijms20122902 PMC6628026

[cre270120-bib-0022] Morishima, H. , J. Washio , J. Kitamura , Y. Shinohara , T. Takahashi , and N. Takahashi . 2017. “Real‐Time Monitoring System for Evaluating the Acid‐Producing Activity of Oral Squamous Cell Carcinoma Cells at Different Environmental pH.” Scientific Reports 7: 10092. 28855722 10.1038/s41598-017-10893-y PMC5577156

[cre270120-bib-0023] Mysak, J. , S. Podzimek , P. Sommerova , et al. 2014. “ *Porphyromonas gingivalis*: Major Periodontopathic Pathogen Overview.” Journal of Immunology Research 2014: 1–8. 10.1155/2014/476068 PMC3984870 24741603

[cre270120-bib-0024] Nakagawa, M. , M. Shirasugi , T. Yamamoto , T. Nakaya , and N. Kanamura . 2021. “Long‐Term Exposure to Butyric Acid Induces Excessive Production of Matrix Metalloproteases in Human Gingival Fibroblasts.” Archives of Oral Biology 123: 105035. 33485112 10.1016/j.archoralbio.2020.105035

[cre270120-bib-0025] Niederman, R. , Y. Buyle‐Bodin , B. Y. Lu , P. Robinson , and C. Naleway . 1997. “Short‐Chain Carboxylic Acid Concentration in Human Gingival Crevicular Fluid.” Journal of Dental Research 76, no. 1: 575–579. 9042080 10.1177/00220345970760010801

[cre270120-bib-0026] Pöllänen, M. T. , D. O. Overman , and J. I. Salonen . 1997. “Bacterial Metabolites Sodium Butyrate and Propionate Inhibit Epithelial Cell Growth *In Vitro* .” Journal of Periodontal Research 32, no. 3: 326–334. 9138199 10.1111/j.1600-0765.1997.tb00541.x

[cre270120-bib-0027] Shinohara, Y. , J. Washio , Y. Kobayashi , Y. Abiko , K. Sasaki , and N. Takahashi . 2021. “Hypoxically Cultured Cells of Oral Squamous Cell Carcinoma Increased Their Glucose Metabolic Activity Under Normoxic Conditions.” PLoS One 16, no. 10: e0254966. 34679081 10.1371/journal.pone.0254966 PMC8535375

[cre270120-bib-0028] Shirasugi, M. , M. Nakagawa , K. Nishioka , T. Yamamoto , T. Nakaya , and N. Kanamura . 2018. “Relationship Between Periodontal Disease and Butyric Acid Produced by Periodontopathic Bacteria.” Inflammation and Regeneration 38: 23. 30574217 10.1186/s41232-018-0081-x PMC6296098

[cre270120-bib-0029] Shirasugi, M. , K. Nishioka , T. Yamamoto , T. Nakaya , and N. Kanamura . 2017. “Normal Human Gingival Fibroblasts Undergo Cytostasis and Apoptosis After Long‐Term Exposure to Butyric Acid.” Biochemical and Biophysical Research Communications 482, no. 4: 1122–1128. 27914813 10.1016/j.bbrc.2016.11.168

[cre270120-bib-0030] Sorkin, B. C. , and R. Niederman . 1998. “Short Chain Carboxylic Acids Decrease Human Gingival Keratinocyte Proliferation and Increase Apoptosis and Necrosis.” Journal of Clinical Periodontology 25, no. 4: 311–315. 9565282 10.1111/j.1600-051x.1998.tb02446.x

[cre270120-bib-0031] Takahashi, N. 2015. “Oral Microbiome Metabolism: From ‘Who Are They?’ To ‘What Are They Doing?” Journal of Dental Research 94, no. 12: 1628–1637. 26377570 10.1177/0022034515606045

[cre270120-bib-0032] Takahashi, N. , T. Sato , and T. Yamada . 2000. “Metabolic Pathways for Cytotoxic End Product Formation From Glutamate‐ and Aspartate‐Containing Peptides by *Porphyromonas gingivalis* .” Journal of Bacteriology 182, no. 17: 4704–4710. 10940008 10.1128/jb.182.17.4704-4710.2000 PMC111344

[cre270120-bib-0033] Takahashl, N. , K. Saito , C. F. Schachtele , and T. Yamada . 1997. “Acid Tolerance and Acid‐Neutralizing Activity of *Porphyromonas gingivalis, Prevotella intermedia* and *Fusobacterium nucleatum* .” Oral Microbiology and Immunology 12, no. 6: 323–328. 9573805 10.1111/j.1399-302x.1997.tb00733.x

[cre270120-bib-0034] Tonetti, M. , C. Eftimiadi , G. Damiani , P. Buffa , D. Buffa , and G. A. Botta . 1987. “Short Chain Fatty Acids Present in Periodontal Pockets May Play a Role in Human Periodontal Diseases.” Journal of Periodontal Research 22, no. 3: 190–191. 2955096 10.1111/j.1600-0765.1987.tb01565.x

[cre270120-bib-0035] Tsuda, H. , K. Ochiai , N. Suzuki , and K. Otsuka . 2010. “Butyrate, a Bacterial Metabolite, Induces Apoptosis and Autophagic Cell Death in Gingival Epithelial Cells.” Journal of Periodontal Research 45, no. 5: 626–634. 20546110 10.1111/j.1600-0765.2010.01277.x

[cre270120-bib-0036] Wu, L. , Z. Luo , Y. Chen , et al. 2023. “Butyrate Inhibits Dendritic Cell Activation and Alleviates Periodontitis.” Journal of Dental Research 102, no. 12: 1326–1336. 37775917 10.1177/00220345231187824

[cre270120-bib-0037] Zheng, T. , F. Lu , P. Wu , Y. Chen , R. Zhang , and X. Li . 2025. “Ferroptosis and Cuproptosis in Periodontitis: Recent Biological Insights and Therapeutic Advances.” Frontiers in Immunology 16: 1526961. 40066457 10.3389/fimmu.2025.1526961 PMC11891063

